# COVID‐19 bimodal clinical and pathological phenotypes

**DOI:** 10.1002/ctm2.648

**Published:** 2022-01-24

**Authors:** Sabrina S. Batah, Maíra N. Benatti, Li Siyuan, Wagner M. Telini, Jamile O. Barboza, Marcelo B. Menezes, Tales R. Nadai, Keyla S. G. Sá, Chirag M. Vaswani, Sahil Gupta, Dario S. Zamboni, Danilo T. Wada, Rodrigo T. Calado, Renê D. R. Oliveira, Paulo Louzada‐Junior, Maria Auxiliadora‐Martins, Flávio P. Veras, Larissa D. Cunha, Thiago M. Cunha, Rodrigo Luppino‐Assad, Marcelo L. Balancin, Sirlei S. Morais, Ronaldo B. Martins, Eurico Arruda, Fernando Chahud, Marcel Koenigkam Santos, Andrea A. Cetlin, Fernando Q. Cunha, Claudia dos Santos, Vera L. Capelozzi, Junya Fukuoka, Rosane Duarte Achcar, Alexandre T. Fabro

**Affiliations:** ^1^ Department of Pathology and Legal Medicine Ribeirão Preto Medical School University of São Paulo São Paulo Brazil; ^2^ Pulmonary Division Department of Internal Medicine Ribeirão Preto Medical School University of São Paulo São Paulo Brazil; ^3^ Department of Surgery Ribeirão Preto Medical School University of São Paulo São Paulo Brazil; ^4^ Hospital Estadual de Bauru São Paulo Brazil; ^5^ Department of Cell and Molecular Biology and Pathogenic Bioagents Ribeirão Preto Medical School University of São Paulo São Paulo Brazil; ^6^ Department of Physiology Temerty Faculty of Medicine University of Toronto Toronto Ontario Canada; ^7^ Keenan Research Centre for Biomedical Science St. Michael's Hospital Toronto Ontario Canada; ^8^ Temerty Faculty of Medicine Institute of Medical Science University of Toronto Toronto Ontario Canada; ^9^ Department of Critical Care Medicine St. Michael's Hospital Toronto Ontario Canada; ^10^ Department of Medical Images Hematology and Oncology Ribeirão Preto Medical School University of São Paulo São Paulo Brazil; ^11^ Division of Clinical Immunology Emergency, Infectious Diseases and Intensive Care Unit, Ribeirão Preto Medical School University of São Paulo São Paulo Brazil; ^12^ Division of Intensive Care Medicine Department of Surgery and Anatomy Ribeirão Preto Medical School University of São Paulo São Paulo Brazil; ^13^ Department of Pharmacology Ribeirão Preto Medical School University of São Paulo São Paulo Brazil; ^14^ Department of Internal Medicine Ribeirão Preto Medical School University of São Paulo São Paulo Brazil; ^15^ Department of Pathology Faculty of Medicine University of São Paulo São Paulo Brazil; ^16^ Interdepartmental Division of Critical Care Medicine University of Toronto Toronto Ontario Canada; ^17^ Department of Pathology Nagasaki University Graduate School of Biomedical Sciences Nagasaki Japan; ^18^ National Jewish Health Department of Medicine Pathology Division Denver Colorado USA

Dear Editor,

For the first time to our knowledge, we describe a bimodal phenotype of coronavirus disease 2019 (COVID‐19) patients provided by clinical and pathological correlation and multidisciplinary discussion, characterized by thrombotic and fibrotic poles (Figure 1).

**FIGURE 1:**
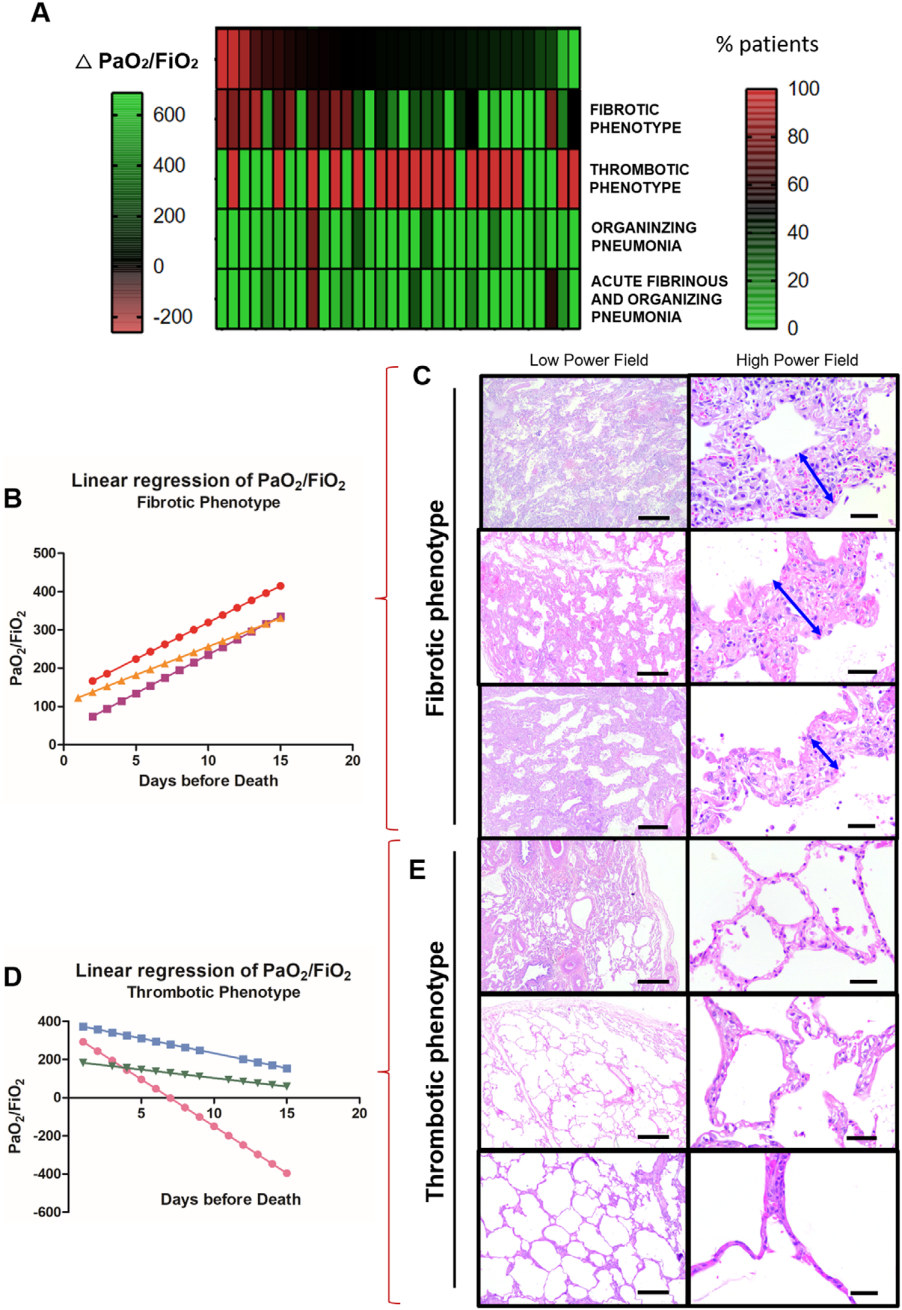
COVID‐19 bimodal clinical and pathological phenotypes. After a multidisciplinary discussion and data integration and analysis, bimodal clinical and pathological phenotypes of COVID‐19 minimally invasive autopsies were discovered by PaO_2_/FiO_2_ linear regression and lung morphology correlation, presented as a gradient (A). The two opposite ends of lung injury were named as (1) Fibrotic phenotype (N = 5) presenting progressive decline in PaO_2_/FiO_2_ ratio (B) with significant alveolar septal thickening by fibrosis (C–double blue arrows) without thrombus formation (C); and (2) Thrombotic Phenotype (N = 10) presenting a progressive increase in PaO_2_/FiO_2_ ratio (D) with recovery of acute/sub‐acute lung injury to or near to normal parenchyma architecture and thrombus formation on vessels (E). Between both phenotypes, a population (N = 32) of non‐bimodal phenotype with different stages of acute, organizing and fibrotic lung injury (A). Scale bar indicates 500 μm in the low power field and 50 μm in high power field (C and E)

Patients with COVID‐19 may present various symptoms leading to different clinical complications and outcomes, ranging from mild to severe.^1^, ^2^ Differences in these outcomes can be attributed to a differential host response to infection with variable viral load, age, gender, comorbidities, genetic and immune background. While different studies have stratified heterogenous COVID‐19 patients based on different strategies,^3^ the characterization of histopathological patterns in association with clinical outcome of patients who died from COVID‐19 remains largely unreported. We hypothesized that COVID‐19 could lead to different lung injury/repair mechanisms related to different clinical and ventilatory manifestations. Therefore, we aimed to assess whether a clinical and pathological correlation could explain the different clinical outcomes among COVID‐19 patients with commonly associated long‐term lung dysfunction.

To answer our question, we performed 47 consecutive COVID‐19 patients minimally invasive autopsies followed by lung morphological analysis and clinical and radiological evaluation (Supporting information Tables S1‐S4). A detailed description of the methodology can be found in the Supporting information. After a multidisciplinary discussion and data integration, we discovered a bimodal clinical and pathological phenotype presented as poles, not groups (Figure 1A; Supporting information Tables S5‐S9): (1) Fibrotic phenotype (N = 5)—characterized by a progressive decline in PaO_2_/FiO_2_ ratio (Figure 1B) with low compliance levels during hospitalization (Supporting information Table S5) and alveolar septal thickening with myxoid fibrosis typical to an organizing phase of diffuse alveolar damage (DAD) (Figure 1C; Supporting information Table S6); and (2) Thrombotic phenotype (N = 10)—characterized by a progressive increase in PaO_2_/FiO_2_ ratio (Figure 1D) with high pulmonary compliance levels during hospitalization (Supporting information Table S5) and recovery of the acute/sub‐acute lung injury or slight distortion of the underlying lung parenchyma architecture with a high frequency of thrombosis (80%) (Figure 1E; Supporting information Table S6). Additionally, although symptoms, signs, demographics, or comorbidities did not correlate to the phenotypes (Supporting information Table S7), d‐dimer and platelets count was higher in these thrombotic patients than fibrotic ones (Supporting information Figure S1A and B and Table S8).

The fibrotic phenotype results from the imbalance between myofibroblastic activation and further deposition/degradation of the collagen and elastic fibers to cause alveolar septal thickening (Figure 2), as reported in other studies.^4^, ^5^ All these features were highlighted in fibrotic phenotype compared to thrombotic one, as follows: (1) higher active myofibroblasts α‐SMA area fraction (Figure 2A and B); (2) higher α‐SMA and MMP‐2 expression (Figure 2C and D); (3) increased extracellular matrix deposition by types I and III collagen fibers by Picrosirius Red staining (Figure 2E and F); and (4) reduced elastic fibers area fraction by Verhöeff staining (Figure 2G and H), correlating negatively to α‐SMA (*r* = −0.57; *p* = 0.035; Supporting information Figure S2A). This dysfunctional alveolar septal thickening in the fibrotic phenotype impairs adequate lung function and gas exchange, which is reflected by the suggestive correlation between collagen fibers deposition and PaO_2_/FiO_2_ ratio (*r* = −0.64; *p* = 0.019; Supporting information Figure S2B and C) and significant negative correlation between compliance and drive pressure (*r* = −0.67; *p* = 0.001; Supporting information Figure 2D). According to other studies,^6^, ^7^ these patients presented a progressive decline in PaO_2_/FiO_2_ ratio (Figure 1B) and low compliance levels during hospitalization (Supporting information Table S5), suggesting a poor clinical outcome. Moreover, fibrotic phenotype patients showed neutrophil extracellular traps to a significantly greater extent throughout lung parenchyma compared to thrombotic phenotype patients (*p* = 0.0004) (Supporting information Figure S3), which is a crucial response during the acute COVID‐19 phase and a potential contributory factor to later fibrotic phase by amplifying the chronic reparative phase.^8^

**FIGURE 2:**
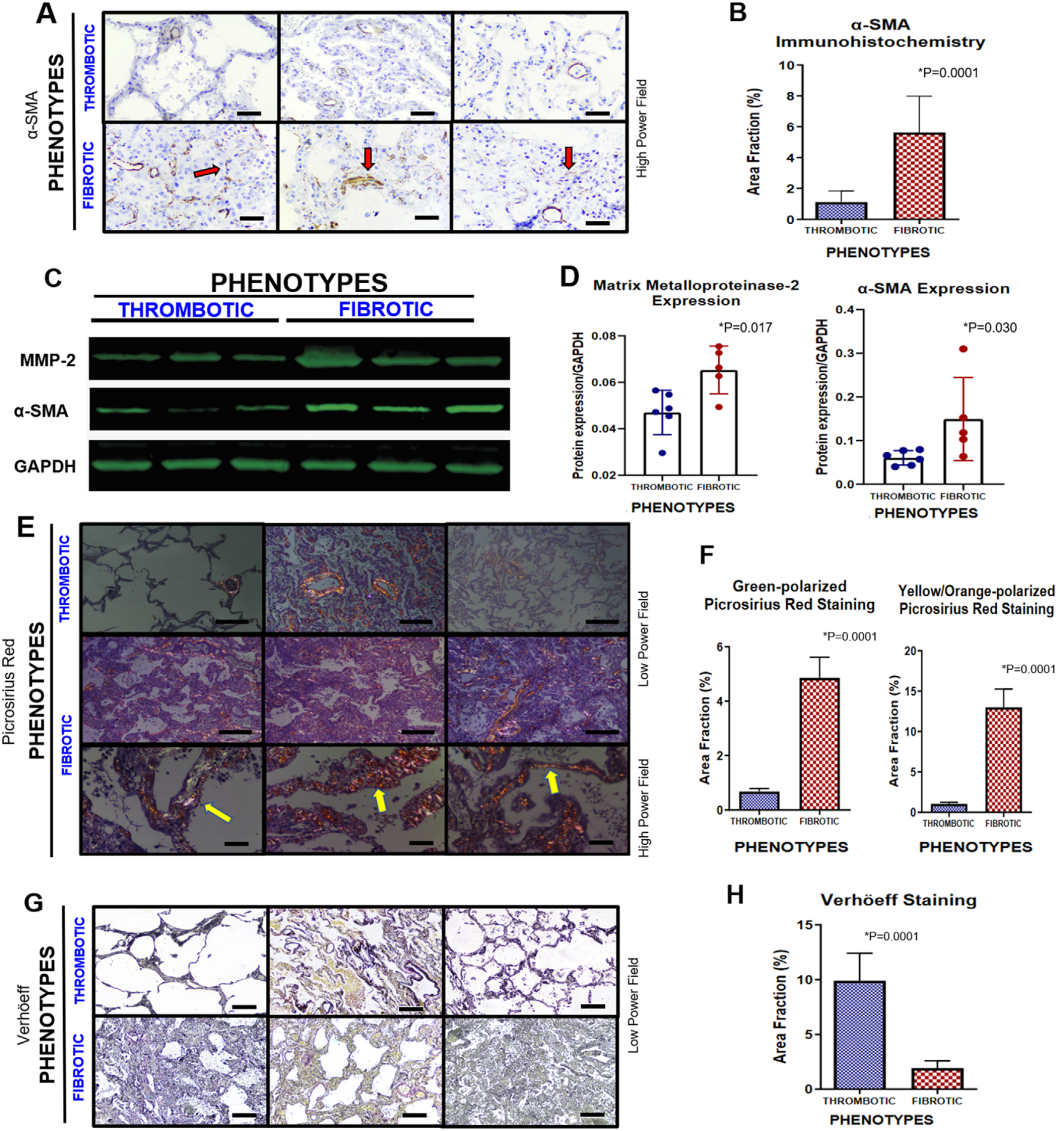
Pulmonary morphological panels of COVID‐19 bimodal phenotypes. Cells expressing α‐SMA by immunohistochemistry were highlighted in fibrotic phenotype compared to thrombotic one (red arrows), probably induced by myofibroblastic activation and neovasculogenesis (A). The α‐SMA expression is confirmed by morphometric analysis (B). Protein expression levels of α‐SMA and MMP‐2 were performed by Western blot (C). GAPDH was used as gene expression control (C–last line). Their quantification was also increased in fibrotic phenotype compared to thrombotic one, confirming the disbalance of production/degradation of extracellular matrix (D). Additionally, extracellular matrix deposition was confirmed by alveolar septal thickening in fibrotic phenotype seen by polarized birefringence of Picrosirius red staining (E–yellow arrow). Morphometric analysis of green and yellow orange polarized collagen fibers by Picrosirius red staining was significantly higher in fibrotic phenotype than thrombotic one (F). Inversely, lung elastic fibers related to elastic tissue capacity were rarefied in fibrotic phenotype by Verhöeff staining compared to thrombotic one (G). This finding was confirmed by morphometric analysis (H). Scale bar indicates 50 μm in high power field (A and E) and 200 μm in low power field (E and G)

Conversely, patients from the thrombotic phenotype showed an unexpected progressive increase in PaO_2_/FiO_2_ ratio (Figure 1D), correlating to d‐dimer levels (*r* = 0.37; *p* = 0.032; Supporting information Figure 2E) and high pulmonary compliance levels (Supporting information Table S5) correlating to elastic fibers (*r* = 0.57; *p* = 0.042; Supporting information Figure S2F), as also surprisingly reported in Gattinoni et al.^9^ This may be justified by the gradual recovering of the acute/sub‐acute lung injury (Figure 1E) with an increased area fraction of elastic fibers (Figure 2G and H). Nevertheless, sudden death occurred, probably due to higher frequency (80%) of pulmonary thromboembolism (Supporting information Table S6), equally described in other study.^10^ As expected, d‐dimer levels and platelet counts were higher in these patients than in fibrotic phenotype subjects (Supporting information Figure S1A and B and Table S8).

Besides those two crucial phenotypes in opposite poles, it is essential to mention that 32 patients (68%) had a mixed pattern of lung injury appearing as a non‐bimodal phenotype, with some overlapping of histopathological findings (Figure 1A). Thus, the pathophysiology of these patients probably represents different stages of viral lung injury with both features of acute/sub‐acute and fibrotic/organizing lung injury at variable degrees.

Regardless of the phenotypes, upon viral insult to the airways and bronchial epithelial cells, severe lymphocytic bronchiolitis occurs, promoting epithelial injury and cell death, eventually resulting in structural disarray by airway‐centered remodeling (Figure 3). The aggressive airway wall infection then spreads to the lung parenchyma, inducing cellular pneumonitis and damaging the alveolar‐capillary barrier (Figure 3, yellow arrow). This could promote DAD, bronchiolocentric alveolar hemorrhage, or fibroblastic plug, called organizing pneumonia (OP), associated or not with fibrin balls, known as acute fibrinous and OP (Figure 3). These pathophysiological processes in COVID‐19 tend to happen more slowly and gradually when compared to H1N1,^11^ around 15 to 20 days, as shown in our cohort (Supporting information Table S1). Therefore, the longer disease progression could give histopathological basis for an organizing phase, with sustained myofibroblastic proliferation, interstitial scarring and parenchymal remodeling, representing the “birth” of fibrosis as a future and possible sequel in post‐COVID‐19 patients, called as the fibrotic phenotype; or gradual recovery of the acute/sub‐acute lung injury associated with thrombosis and unexpected sudden death, called as thrombotic phenotype (Figures 1 and 3).

**FIGURE 3:**
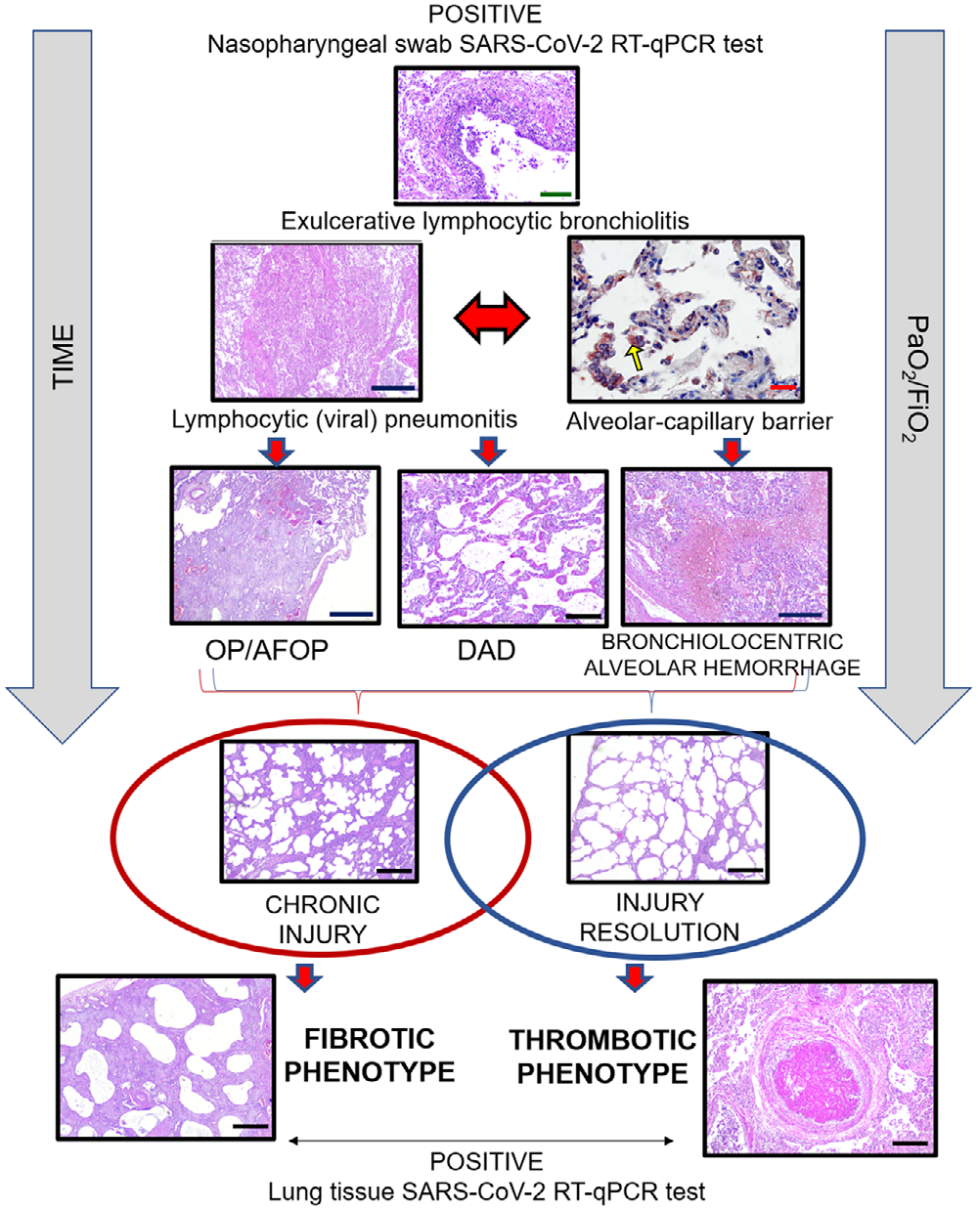
COVID‐19 lung pathophysiology. The viral infection starts in the lungs first by reaching the airways and infecting bronchial epithelial cells. Then, the pathophysiological processes in response to the viral attack cause an exulcerating lymphocytic bronchiolitis, followed by lymphocytic (viral) pneumonitis. Consequently, the second wave of infection overspreads through the lung parenchyma, inducing cellular pneumonitis, which injures the alveolo‐capillar barriers near distal airways, highlighted by immunohistochemistry with anti‐SARS‐CoV‐2 (yellow arrow). Then, fibroplastic balls (organizing pneumonia–OP) and fibrin balls (acute fibrinous and organizing pneumonia–AFOP), diffuse alveolar damaged (DAD) and bronchiolocentric alveolar hemorrhage may occur in the injured lung tissue. Over the time of viral infection and mechanical ventilation, two different repair processes can occur depending on the resolution or progression of the injury, coinciding with the bimodal clinic‐pathological phenotype: (1) Chronic injury with fibrotic phenotype; or (2) Gradual injury resolution with thrombotic phenotype. Scale bars indicate: 500 μm (blue), 200 μm (black), 100 μm (green) and 50 μm (red)

Consequently, the reported sudden deaths of COVID‐19 patients in clinical improvement and the description of post‐COVID‐19 tomographic fibrotic changes may be justified respectively by thrombotic and fibrotic phenotypes.^12^ Two COVID‐19 patients’ follow‐up with transbronchial biopsies, performed at 3 to 5 months post‐infection, demonstrated lung parenchyma remodeling with alveolar septal thickening by dense fibrosis (Supporting information Figure S4).

The present study presents a unique histopathological analysis of 47 COVID‐19 autopsy cases with clinical and pathological correlation. We cannot disregard some limitations due to our sample size, as 47 cases are too few to establish ground rules for COVID‐19 outcomes. Nevertheless, our samples are exceptional and should be valued. In conclusion, we believe that the categorization of patients based on these two phenotypes can be used to develop prognostic tools and potential therapies since the PaO_2_/FiO_2_ ratio and d‐dimer correlate with the underlying fibrotic or thrombotic pathophysiologic process, which may indicate the possible clinical outcome of the patient.

## CONFLICT OF INTEREST

The authors declare no conflict of interest.

## STUDY APPROVAL

The procedures followed in the study were approved by the National Ethics Committee–Brazil (CAAE: 32475220.5.0000.5440). The written informed consent was waived.

## AVAILABILITY OF METHODOLOGY

Additional supporting information for methodology may be found online in the Supporting File.

## Supporting information

Supporting Information
