# Supplementary material for: COVID‐19 bimodal clinical and pathological phenotypes
Source: Clin Transl Med. 2022 Jan 24;12(1):e648. doi: 10.1002/ctm2.648 (PMC8787021; doi:10.1002/ctm2.648)
Supplement: Supplementary file 1 — Supporting Information [file CTM2-12-e648-s001.docx]

**COVID-19 BIMODAL CLINICAL AND PATHOLOGICAL PHENOTYPES**

**Supplementary Material**

*Sabrina S Batah^1^; Maíra N Benatti^2^; Li Siyuan^3^; Wagner M Telini^1^;* *Jamile Barboza^1^; Marcelo B Menezes^2^;* Tales *R* Nadai^4^; *Keyla S G Sá^5^;* *Chirag M. Vaswani^6,7^; Sahil Gupta^7,8,9^ Dario S Zamboni^5^; Danilo T Wada^10^;* [*Rodrigo T Calado*](https://rio.fmrp.usp.br/perfil/rtcalado/)*^10^; Renê D R Oliveira^11^; Paulo Louzada-Junior^11^; Maria Auxiliadora-Martins^12^; Flávio P Veras^13^; Larissa D Cunha^5^; Thiago M Cunha^13^; Rodrigo Luppino-Assad^14;^ Marcelo L Balancin^15^; Sirlei S Morais^1^; Ronaldo B Martins^5^; Eurico Arruda^5^; Fernando Chahud^1^; Marcel Koenigkam-Santos^10^; Andrea A Cetlin^2^; Fernando Q Cunha^13^; Claudia dos Santos^7,16^; Vera L Capelozzi^15^; Junya Fukuoka^17;^ Rosane Duarte Achcar^18ƺ^; Alexandre T Fabro^1^**

^1^Department of Pathology and Legal Medicine, Ribeirão Preto Medical School, University of São Paulo, Brazil; ^2^Pulmonary Division, Department of Internal Medicine, Ribeirão Preto Medical School, University of São Paulo, Brazil; ^3^Department of Surgery, Ribeirão Preto Medical School, University of São Paulo, Brazil; ^4^Hospital Estadual de Bauru, Brazil; ^5^Department of Cell and Molecular Biology and Pathogenic Bioagents, Ribeirão Preto Medical School, University of São Paulo, Brazil; ^6^Department of Physiology, Temerty Faculty of Medicine, University of Toronto, Toronto, ON, CA; ^7^Keenan Research Centre for Biomedical Science, St. Michael’s Hospital, Toronto, ON, CA; ^8^Institute of Medical Science, Temerty Faculty of Medicine, University of Toronto, 1 King’s College Circle, Toronto, ON, CA; ^9^Department of Critical Care Medicine, St. Michael’s Hospital, Toronto, ON, CA; ^10^Department of Medical Images, Hematology and Oncology, Ribeirão Preto Medical School, University of São Paulo, Brazil; ^11^Division of Clinical Immunology, Emergency, Infectious Diseases and Intensive Care Unit, Ribeirão Preto Medical School, University of São Paulo, Brazil; ^12^Division of Intensive Care Medicine, Department of Surgery and Anatomy, Ribeirão Preto Medical School, University of São Paulo; Brazil; ^13^Department of Pharmacology, Ribeirão Preto Medical School, University of São Paulo, Brazil; ^14^Department of Internal Medicine, Ribeirão Preto Medical School, University of São Paulo, Brazil; ^15^Department of Pathology, Faculty of Medicine, University of São Paulo, Brazil; ^16^Interdepartmental Division of Critical Care Medicine, University of Toronto, Toronto, ON, Canada; ^17^Department of Pathology, Nagasaki University Graduate School of Biomedical Sciences, Nagasaki, Japan; ^18^National Jewish Health, Department of Medicine, Pathology Division, Denver, Colorado, USA.

*^ƺ^co-senior author*

***Corresponding author**

**METHODS**

**Study Design**

Forty-seven consecutive COVID-19 patients with positive nasopharyngeal swab for SARS-CoV-2 by reverse transcription polymerase chain reaction (RT-PCR) were considered eligible for this study. Modified minimally invasive autopsy (MIA)[^1^](#_ENREF_1) was performed at University Hospital of Ribeirão Preto Medical School, University of São Paulo, Ribeirão Preto, SP, Brazil – HCFMRP/USP from May to July, 2020. Briefly, all MIAs were performed at bedside through post-mortem surgical lung biopsy within 1 hour of death by a 3 cm incision on the anterior side of the chest between the fourth and fifth ribs. A matching 14-gauge cutting needle (Magnum Needles, Bard) and a biopsy gun (Magnum, Bard) also were used. Two post-COVID-19 biopsies cases were included. This study was approved by the local Research Ethics Committee and written informed consent was waived.

**Data Collection**

Electronic medical record of all patients (N=47) enrolled in this study was collected. Demographic, clinical data, symptoms, drugs, treatments prescribed and laboratory tests were recorded thoroughly. Laboratory tests were collected and identified as: 1) Admission tests: up to the first 48 hours of admission; and 2) Daily tests: on the fifth, third and first day before death.

In order to better understand patient’s outcome, mechanical ventilation parameters of each individual patient were recorded. All daily change of these variables (from the first to the last day of mechanical ventilation until death) were registered and the means recorded for the last day before death of each individual. In addition, PaO_2_/FiO_2_ linear regression was performed with all available values. Then, to determine which patients had changes of lung function, we calculated the larger and smaller angles from the lines of PaO_2_/FiO_2_ linear regression. Finally, chest X-ray (CXR) and high-resolution computed tomography (HRCT) images of all patients were reviewed and evaluated by two specialized pulmonary radiologists (MKS and DTW). Unfortunately, only 15 patients had HRCT scan, preventing further analyses.

**Histological Evaluation**

Lung tissue samples were fixed in 10% buffered formalin, for at least 48 h. Paraffin-embedded sections of 3-μm thickness were stained with Haematoxylin and Eosin, Picrosirius red (Abcam, ab150681) for collagen fibers identification and Verhoeff (Abcam, ab150667) for elastic fibers identification, according to local protocol. Immunohistochemistry for anti-alpha smooth muscle actin (α-SMA) (Abcam, ab5694) and anti-SARS-CoV-2 polyclonal antibody, developed by our group for in situ detection of SARS-CoV-2, were performed in paraffin-embedded sections of 3-μm thickness, following our lab protocol[^2^](#_ENREF_2). Histological evaluation was performed by specialized pulmonary pathologists (MLB, VLC, ATF) blinded to clinical history. For all patients, histopathological features were assessed as present or absent. Cut-offs of area involvement were determined for fibrotic phenotype, OP and AFOP, while thrombus formation was considered present or absent.

**Histomorphometry**

Histochemistry and immunohistochemistry stains were quantified by morphometry. The images were captured with a digital camera on microscope (Novel L3000 LED) and analyzed using Image Pro Plus 7 software. Quantification followed the morphometric standards established by American Thoracic Society and European Thoracic Society (ATS/ERS)[^3^](#_ENREF_3). Positive α-sma cells were evaluated in ten different, randomly selected high-power fields of the lungs. At 400x magnification, the number of positive cells in each field was calculated according to the number of points hitting positive cells as a proportion of the total grid area. The density of collagen and elastic fibers was measured in the lung parenchyma in ten randomly selected microscopic fields at a magnification of ×200. The threshold for collagen fibers was established for all slides after the contrast was enhanced to the point at which the fibers were easily identified as green or orange bands. The density of the collagen and elastic fibers was expressed as the ratio between the measured fibers divided by the total area studied ×100.

**Immunofluorescence Analysis**

Neutrophils and neutrophil-derived extracellular traps (NETs) were identified and quantified by immunofluorescence with anti-myeloperoxidase (MPO) (Abcam, ab25989), anti-citrullinated histone H3 (H3Cit) (Abcam, ab5103) and DAPI (Life Technologies, D1306). The staining and quantification were performed as our lab protocol[^4^](#_ENREF_4).

**Western Blotting Analysis**

Protein extraction were performed for all fresh lung samples with RIPA buffer (10 mM Tris-HCl, pH 7.4, 1 mM EDTA, 150 mM NaCl, 1% Nonidet P-40, 1% deoxycholate and 0.1% SDS) in the presence of protease inhibitor cocktail (Roche). Then, the lysate and supernatant were solubilized in a heated Laemmli buffer, added to the SDS-PAGE and transferred into a 0.22-μm nitrocellulose membrane (GE Healthcare Biosciences, cat. 10600002). The membranes were blocked with Tris-buffered saline (TBS) with 0.01% Tween-20 and 5% skimmed milk powder and incubated with anti-α-SMA (Abcam, ab7817), anti-matrix metalloproteinase-2 (anti-MMP-2) (Abcam, ab97779) and anti-glyceraldehyde-3-phosphate dehydrogenase (anti-GAPDH) (Abcam, ab9485). The proteins were detected and quantified with ChemiDoc Imaging Systems (Bio-Rad) and the results were normalized by GAPDH expression.

**Real-Time Polymerase Chain Reaction for Viral RNA**

Total RNA from fresh lung tissue of all patients was obtained using QIAzol Lysis Reagent (Qiagen). Reverse transcriptase was performed using SuperScript IV reverse transcriptase (Invitrogen, Carlsbad, USA) according to manufacturer’s instructions and our standard protocol^[5](#_ENREF_5)^. RT-PCR for detection of SARS-CoV-2 nucleocapsid (N2) and envelope (E) viral proteins was performed on Step One Plus Real-Time PCR System (Applied Biosystems,Grand Island, NY) with TaqMan probes (Applied Biosystems). Both primers were designed by ThermoFisher Scientific from N2 and E genes sequences developed by Centers for Disease Control and Prevention (CDC) of Atlanta (USA)[^6^](#_ENREF_6) and Charité Germany[^7^](#_ENREF_7), respectively. Samples were tested in triplicate and cycle threshold values less than 44 were considered positive.

**Statistical Analysis**

Statistical analysis was performed with SPSS v.13.0 0 software (SPSS, Inc., Chicago, IL, 2004). The data was evaluated using T test. Furthermore, nonparametric correlation (Spearman) was also performed with all collected data. Linear regression was also performed for these patients with all available PaO_2_/FiO_2_ values. Data are expressed as mean ± standard deviation and the p value less than 0.05 was be considered statistically significant.

**REFERENCES**

1. Weustink AC, Hunink MM, Van Dijke CF, Renken NS, Krestin GP, Oosterhuis JW. Minimally invasive autopsy: an alternative to conventional autopsy? *Radiology.* 2009;250(3):897-904.

2. Pontelli MC, Castro IA, Martins RB, et al. Infection of human lymphomononuclear cells by SARS-CoV-2. *bioRxiv.* 2020:2020.2007.2028.225912.

3. Hsia CC, Hyde DM, Ochs M, Weibel ER, Structure AEJTFoQAoL. An official research policy statement of the American Thoracic Society/European Respiratory Society: standards for quantitative assessment of lung structure. *Am J Respir Crit Care Med.* Feb 15 2010;181(4):394-418.

4. Veras FP, Pontelli MC, Silva CM, et al. SARS-CoV-2-triggered neutrophil extracellular traps mediate COVID-19 pathology. *The Journal of experimental medicine.* Dec 7 2020;217(12).

5. Pontelli MC, Castro IA, Martins RB, et al. Infection of human lymphomononuclear cells by SARS-CoV-2. *bioRxiv.* 2020:2020.2007.2028.225912.

6. Control CfD, Prevention. CDC 2019-novel coronavirus (2019-nCoV) real-time RT-PCR diagnostic panel. 2020.

7. Corman VM, Landt O, Kaiser M, et al. Detection of 2019 novel coronavirus (2019-nCoV) by real-time RT-PCR. *Euro surveillance : bulletin Europeen sur les maladies transmissibles = European communicable disease bulletin.* Jan 2020;25(3).

**SUPPLEMENTARY TABLES**

**Supplementary** **Table S1 – COVID-19 Clinical Data.**

| **All patients (N=47)** | | |
| --- | --- | --- |
| **DEATH AS A STARTING POINT** | | |
|  | **Death after onset of COVID-19 symptoms (days)*** | 18.90 ± 11 |
|  | **Hospitalization (days)*** | 14.43 ± 10.13 |
| **RT-PCR** | |  |
|  | **N2 gene (copies/mg)*** | 8.81x10^6^ ± 26.3x10^6^ |
|  | **E gene (copies/mg)*** | 2.83x10^8^± 7.65x10^8^ |
| **DEMOGRAPHICS** | | |
|  | **Gender (M;F)** | 24;23 |
|  | **Age (yrs)*** | 67.83 ± 15.14 |
|  | **Height (cm)*** | 1.68 ± 0.10 |
|  | **Weight (kg)*** | 87.46 ± 28.53 |
|  | **Body mass index (Kg/m²)*** | 31.19 ± 9.08 |
| **COMORBIDITIES** | | |
|  | **Systemic arterial hypertension (%)** | 26 (55.3) |
|  | **Obesity (%)** | 17 (36.2) |
|  | **Smokers (%)** | 14 (29.8) |
|  | **Diabetes (%)** | 10 (21.3) |
|  | **Chronic cardiovascular disease (%)** | 10 (21.3) |
|  | **Chronic respiratory disease (%)** | 10 (21.3) |
|  | **Chronic renal disease (%)** | 7 (14.9) |
|  | **Alcoholism (%)** | 4 (8.5) |
|  | **Others (%)** | 40 (85.1) |
| **FIRST SYMPTOMS AND SIGNS IDENTIFIED** | |  |
|  | **Cough (%)** | 24 (51.1) |
|  | **Fever (%)** | 16 (34.0) |
|  | **Dyspnea (%)** | 17 (36.2) |
|  | **Flu-like symptoms (%)** | 5 (10.6) |
|  | **Myalgia (%)** | 10 (21.3) |
| **SYMPTOMS AND SIGNS BEFORE ADMISSION** | | |
|  | **Cough (%)** | 16 (34.0) |
|  | **Fever (%)** | 9 (19.1) |
|  | **Dyspnea (%)** | 31 (66.0) |
|  | **Flu-like symptoms (%)** | 2 (4.3) |
|  | **Myalgia (%)** | 4 (8.5) |
| **SYMPTOMS AND SIGNS ON ADMISSION** | | |
|  | **Cough (%)** | 18 (38.3) |
|  | **Fever (%)** | 5 (10.6) |
|  | **Dyspnea (%)** | 37 (78.7) |
|  | **Flu-like symptoms (%)** | 0 (0.0) |
|  | **Myalgia (%)** | 0 (0.0) |
| **SYMPTOMS AND SIGNS DURING HOSPITALIZATION** | |  |
|  | **Cough (%)** | 18 (38.3) |
|  | **Fever (%)** | 14 (29.8) |
|  | **Dyspnea (%)** | 19 (40.4) |
|  | **Flu-like symptoms (%)** | 0 (0.0) |
|  | **Myalgia (%)** | 0 (0.0) |
| **NEUROLOGICAL SYMPTOMS** | |  |
|  | **Neurological symptoms (%)** | 7 (14.9) |
|  | **Headache (%)** | 4 (8.5) |
|  | **Dysgeusia (%)** | 3 (6.4) |
|  | **Anosmia (%)** | 5 (10.6) |
| **CLINICAL COMPLICATIONS** | |  |
|  | **Septic shock (%)** | 29 (61.7) |
|  | **Acute kidney injury (%)** | 24 (51.1) |
|  | **Acute respiratory distress syndrome (%)** | 21 (44.7) |
|  | **Myocardial infarction (%)** | 12 (25.5) |
|  | **Congestive heart failure (%)** | 4 (8.5) |
| **MAIN MEDICATIONS** | | |
|  | **Anticoagulant (%)** | 47 (100.0) |
|  | **Steroids (%)** | 38 (80.9) |
|  | **Vasopressor (%)** | 37 (78.7) |
|  | **Chloroquine/Hydroxychloroquine (%)** | 2 (4.3) |
| **LABORATORY TESTS: ADMISSION (Highest value)** | |  |
|  | **Lactate dehydrogenase (U/L)*** | 783.12 ± 686.64 |
|  | **C-reactive protein (mg/L)*** | 13.87 ± 8.72 |
|  | **D-dimer (mg/L)*** | 4.22 ± 3.85 |
| **TREATMENTS** | | |
|  | **Prone positioning (%)** | 28 (59.6) |
|  | **Neuromuscular blockade (%)** | 29 (61.7) |
| **MECHANICAL VENTILATION PARAMETERS** | | |
|  | **Time of death after mechanical ventilation (days)*** | 14.53 ± 10.70 |
|  | **Mechanical Ventilation (%)** | 36 (76.6) |
|  | **PaO_2_/FiO_2_ ratio*** † | 172.22 ± 90.64 |
|  | **Drive pressure cmH_2_O *** † | 13.70 ± 4.42 |
|  | **Plateau pressure cmH_2_O *** † | 22.85 ± 4.18 |
|  | **PICO cmH_2_O*** † | 29.50 ± 8.18 |
|  | **PEEP cmH_2_O *** † | 9.27 ± 2.21 |
|  | **Compliance ml/cmH2O*** † | 31.53 ± 15.53 |

**Note:** * mean ± standard deviation; (%) percentage; † Last measure before death.

**Supplementary** **Table S2 - COVID 19 Radiological Features.**

| **All patients (N=47)** | | |
| --- | --- | --- |
| **CHEST XR** | | |
|  | **Ground-glass opacity (%)** | 47 (100.0) |
|  | **Edema (%)** | 8 (17.0) |
|  | **Pulmonary consolidation (%)** | 9 (19.1) |
|  | **Pulmonary congestion (%)** | 3 (6.4) |
|  | **Pleural effusion (%)** | 7 (14.9) |
|  | **Interstitial opacities (%)** | 23 (48.9) |
|  | **Cardiomegaly (%)** | 31 (65.9) |
|  |  |  |
| **15 patients** | | |
| **CHEST CT** | | |
|  | **Crazy-paving (%)** | 5 (33.3) |
|  | **Consolidations (%)** | 13 (86.7) |
|  | **Architectural distortion (%)** | 8 (53.3) |
|  | **Traction bronchiectasis (%)** | 6 (40.0) |
|  | **Honeycombing (%)** | 1 (6.7) |
|  | **Pleural effusion (%)** | 9 (60.0) |

**Note:** (%) percentage.

**Supplementary** **Table S3 - COVID-19 Laboratory Tests.**

| **All patients (N=47)** | | | | | | | |  |
| --- | --- | --- | --- | --- | --- | --- | --- | --- |
| **LABORATORY EXAMS** | | **Highest value** | **One day**  **before death** | **Three days before death** | | **Five days before death** |  |  |
|  | **White cell count (10³/µL)*** | 21.77 ± 11.35 | 21.85 ± 12.29 | 17.14 ± 8.82 | 15.78 ± 7.46 | | |  |
|  | **Hemoglobin (g/dL)*** | 11.06 ± 1.93 | 9.62 ± 2.07 | 10.40 ± 2.29 | 10.61 ± 1.85 | | |  |
|  | **Sodium (mmol/L)*** | 140.8 ± 7.46 | 137.88 ± 7.79 | 139.43 ± 6.99 | 139.57 ± 6.86 | | |  |
|  | **Potassium (mmol/L)*** | 5.12 ± 1.27 | 4.94 ± 1.08 | 4.65 ± 0.97 | 1.66 ± 1.27 | | |  |
|  | **Creatinine (mg/dL)*** | 2.45 ± 1.51 | 2.41 ± 1.45 | 2.13 ± 1.56 | 2.12 ± 1.31 | | |  |
|  | **Lymphocytes (10^3/μl)*** | 1.49 ± 1.14 | 1.37 ± 1.18 | 1.12 ± 0.87 | 1.04 ± 0.86 | | |  |
|  | **Platelets (10^3/μl)*** | 287.36 ± 143.93 | 264.05 ± 152.44 | 253.36 ± 113.81 | | 266.15 ± 130.50 | | |
|  | **Neutrophils (10^3/μl)*** | 18.40 ± 9.31 | 18.60 ± 9.53 | 15.14 ± 7.61 | 14.33 ± 6.63 | | |  |

**Note:** * mean ± standard deviation.

**Supplementary** **Table S4 - COVID19 Minimally Invasive Autopsy Findings.**

| **All patients (N=47)** | | |
| --- | --- | --- |
| **HISTOLOGIC FEATURES** | | |
|  | **Interstitial fibrosis (%)** | 41 (87.2) |
|  | **Organizing pneumonia (%)** | 21 (44.7) |
|  | **Acute fibrinous organizing pneumonia (%)** | 33 (70.2) |
|  | **Thrombus formation (%)** | 25 (53.2) |
|  | **Viral cytopathic effect (%)** | 24 (51.1) |
|  | **Squamous metaplasia (%)** | 13 (27.7) |
|  | **Alveolar hemorrhage (%)** | 29 (61.7) |
|  | **Hyaline membrane (%)** | 22 (46.8) |
|  | **Hyalinization (%)** | 18 (38.3) |
|  | **Intra-alveolar edema (%)** | 6 (12.8) |
|  | **Neutrophilic infiltrate (%)** | 8 (17.0) |
|  | **Occult lung cancer (%)** | 17 (36.2) |
|  | **Bronchiolization (%)** | 21 (44.7) |
|  | **Bronchiectasis** | 24 (51.1) |
|  | **Normal pulmonary parenchyma** | 41 (87.2) |
|  | **Pneumocyte desquamation** | 20 (42.6) |
|  | **Cellular bronchiolitis (%)** | 3 (6.4) |
|  | **Pleural thickening (%)** | 8 (17.0) |

**Note:** (%) percentage.

**Supplementary** **Table S5 - Bimodal Clinical and Pathological Phenotypes: Mechanical Ventilation Data.**

| **MECHANICAL VENTILATION PARAMETERS** | **Thrombotic Phenotype** | **Fibrotic Phenotype** | **P** |
| --- | --- | --- | --- |
| **Time of death after mechanical ventilation (days)*** | 11.4 ± 5.97 | 10.0 ± 4.53 | 0.65 |
| **Mechanical Ventilation (%)** | 10 (100.0) | 5 (100.0) | 0.99 |
| **△ PaO_2_/FiO_2_ *** | 141.0 ± 201.0 | -169.50 ± 99.0 | 0.01 |
| **PaO_2_/FiO_2_ ratio*** † | 198.39 ± 90.25 | 101.16 ± 39.31 | 0.01 |
| **Drive pressure cmH_2_O*** † | 12.22 ± 3.53 | 18.08 ± 4.70 | 0.06 |
| **Plateau pressure cmH_2_O*** † | 21.38 ± 4.0 | 25.75 ± 3.30 | 0.12 |
| **PICO cmH_2_O*** † | 25.3 ± 10.0 | 31.0 ± 5.57 | 0.57 |
| **PEEP cmH_2_O*** † | 9.78 ± 1.56 | 8.50 ± 1.91 | 0.31 |
| **Compliance ml/cmH_2_O*** † | 41.42 ± 16.32 | 19.80 ± 6.17 | 0.02 |

**Note:** * mean ± standard deviation; (%) percentage; △ Variation between the last (before death) and first (onset of mechanical ventilation) value recorded for PaO_2_/FiO_2_; † Last measure before death

**Supplementary** **Table S6 - Bimodal Clinical and Pathological Phenotypes: Histopathological Findings in Minimally Invasive Autopsy**

| **HISTOLOGICAL FEATURES** | **Thrombotic Phenotype** | **Fibrotic Phenotype** | **P** |
| --- | --- | --- | --- |
| **Fibrotic septal thickening (>20% area)** | 4 (40.0) | 5 (100.0) | 0.01 |
| **Organizing pneumonia (>5% area)** | 2 (20.0) | 0 (0.0) | 0.52 |
| **Acute fibrinous and organizing pneumonia (>5% area)** | 1 (10.0) | 0 (0.0) | 0.99 |
| **Thrombus formation (%)** | 8 (80.0) | 2 (40.0) | 0.25 |

**Note:** (%) percentage.

**Supplementary** **Table S7 – Bimodal COVID-19 Phenotypes: Demographical and Clinical Data.**

| **DEMOGRAPHIC AND CLINICAL DATA** | | **Thrombotic Phenotype** | **Fibrotic Phenotype** | | **P** |  |  |  |
| --- | --- | --- | --- | --- | --- | --- | --- | --- |
| **DEATH AS A STARTING POINT** | | | |  |  | |  |  |
|  | **Time of death from onset of COVID-19 symptoms (days)*** | | 18.30 ± 4.30 | | 18.00 ± 6.12 | 0.92 | | |
|  | **Hospitalization length (days)*** | | 14.10 ± 6.40 | | 13.60 ± 4.22 | 0.88 | | |
| **RT-PCR** | | | |  |  | |  |  |
|  | **N2 gene (copies/mg)** | | 1.21x10^5^ ± 2.64x10^5^ | | 3.92x10^6^ ± 8.49x10^6^ | 0.09 | | |
|  | **E gene (copies/mg)** | | 2.06x10^7^ ± 3.59x10^7^ | | 8.19x10^7^ ± 1.61x10^8^ | 0.61 | | |
| **Demographics** | | | |  |  | |  |  |
|  | **Gender (M;F)** | | 5;5 | | 1;4 | 0.58 | | |
|  | **Age (yrs)*** | | 64.60 ± 12.25 | | 70.60 ± 7.09 | 0.83 | | |
|  | **Height (cm)*** | | 1.69 ± 0.07 | | 1.70 ± 0.07 | 0.96 | | |
|  | **Weight (kg)*** | | 93.88 ± 31.84 | | 82.67 ± 11.24 | 0.81 | | |
|  | **Body mass index (Kg/m²)*** | | 32.61 ± 9.16 | | 28.87 ± 5.93 | 0.63 | | |
| **COMORBIDITIES** | | | | |  | |  |  |
|  | **Systemic arterial hypertension (%)** | | 7 (70.0) | | 3 (60.0) | 0.99 | | |
|  | **Obesity (%)** | | 4 (40.0) | | 2 (40.0) | 0.99 | | |
|  | **Smokers (%)** | | 1 (10.0) | | 3 (60.0) | 0.07 | | |
|  | **Diabetes (%)** | | 6 (60.0) | | 4 (80.0) | 0.60 | | |
|  | **Chronic cardiovascular disease (%)** | | 3 (30.0) | | 0 (0.0) | 0.50 | | |
|  | **Chronic respiratory disease (%)** | | 3 (30.0) | | 0 (0.0) | 0.50 | | |
|  | **Chronic renal disease (%)** | | 2 (20.0) | | 1 (20.0) | 0.99 | | |
|  | **Alcoholism (%)** | | 1 (10.0) | | 0 (0.0) | 0.99 | | |
|  | **Others (%)** | | 9 (90.0) | | 4 (80.0) | 0.99 | | |
| **FIRST SYMPTOMS AND SIGNS IDENTIFIED** | | | |  |  | |  |  |
|  | **Cough (%)** | | 4 (40.0) | | 4 (80.0) | 0.28 | | |
|  | **Fever (%)** | | 3 (30.0) | | 2 (40.0) | 0.99 | | |
|  | **Dyspnea (%)** | | 2 (20.0) | | 1 (20.0) | 0.99 | | |
|  | **Flu-like symptoms (%)** | | 2 (20.0) | | 0 (0.0) | 0.52 | | |
|  | **Myalgia (%)** | | 4 (40.0) | | 2 (40.0) | 0.99 | | |
| **SYMPTOMS AND SIGNS BEFORE ADMISSION** | | | | |  | |  |  |
|  | **Cough (%)** | | 1 (10.0) | | 1 (20.0) | 0.99 | | |
|  | **Fever (%)** | | 1 (10.0) | | 1 (20.0) | 0.99 | | |
|  | **Dyspnea (%)** | | 6 (60.0) | | 2 (40.0) | 0.60 | | |
|  | **Flu-like symptoms (%)** | | 1 (10.0) | | 0 (0.0) | 0.99 | | |
|  | **Myalgia (%)** | | 1 (10.0) | | 0 (0.0) | 0.99 | | |
| **SYMPTOMS AND SIGNS ON ADMISSION** | | | | |  | |  |  |
|  | **Cough (%)** | | 1 (10.0) | | 1 (20.0) | 0.99 | | |
|  | **Fever (%)** | | 1 (10.0) | | 0 (0.0) | 0.99 | | |
|  | **Dyspnea (%)** | | 10 (100.0) | | 4 (80.0) | 0.33 | | |
|  | **Flu-like symptoms (%)** | | 0 (0.0) | | 0 (0.0) | 0.99 | | |
|  | **Myalgia (%)** | | 0 (0.0) | | 0 (0.0) | 0.99 | | |
| **SYMPTOMS AND SIGNS DURING HOSPITALIZATION** | | | |  |  | |  |  |
|  | **Cough (%)** | | 4 (40.0) | | 2 (20.0) | 0.99 | | |
|  | **Fever (%)** | | 6 (60.0) | | 3 (60.0) | 0.99 | | |
|  | **Dyspnea (%)** | | 3 (30.0) | | 2 (20.0) | 0.99 | | |
|  | **Flu-like symptoms (%)** | | 0 (0.0) | | 0 (0.0) | 0.99 | | |
|  | **Myalgia (%)** | | 0 (0.0) | | 0 (0.0) | 0.99 | | |
| **NEUROLOGICAL SYMPTOMS** | | | |  |  | |  |  |
|  | **Neurological symptoms (%)** | | 2 (20.0) | | 0 (0.0) | 0.52 | | |
|  | **Headache (%)** | | 1 (10.0) | | 0 (0.0) | 0.99 | | |
|  | **Dysgeusia (%)** | | 0 (0.0) | | 0 (0.0) | 0.99 | | |
|  | **Anosmia (%)** | | 2 (20.0) | | 0 (0.0) | 0.52 | | |
| **CLINICAL COMPLICATIONS** | | | |  |  | |  |  |
|  | **Septic shock (%)** | | 8 (80.0) | | 4 (80.0) | 0.99 | | |
|  | **Acute kidney injury (%)** | | 8 (80.0) | | 4 (80.0) | 0.99 | | |
|  | **Acute respiratory distress syndrome (%)** | | 4 (40.0) | | 5 (100.0) | 0.88 | | |
|  | **Myocardial infarction (%)** | | 4 (40.0) | | 1 (20.0) | 0.60 | | |
|  | **Congestive heart failure (%)** | | 1 (10.0) | | 0 (0.0) | 0.99 | | |
| **MAIN MEDICATIONS** | | | |  |  | |  |  |
|  | **Anticoagulant (%)** | | 10 (100.0) | | 5 (100.0) | 0.99 | | |
|  | **Steroids (%)** | | 9 (90.0) | | 5 (100.0) | 0.99 | | |
|  | **Vasopressor (%)** | | 10 (100.0) | | 4 (80.0) | 0.33 | | |
|  | **Chloroquine/Hydroxychloroquine (%)** | | 1 (10.0) | | 0 (0.0) | 0.99 | | |
| **TREATMENTS** | | | |  |  | |  |  |
|  | **Prone positioning (%)** | | 10 (100.0) | | 3 (60.0) | 0.95 | | |
|  | **Neuromuscular blockade (%)** | | 10 (100.0) | | 4 (80.0) | 0.33 | | |

**Note:** * mean ± standard deviation; (%) percentage

**Supplementary** **Table S8 – Bimodal COVID-19 Phenotypes: Laboratory Data.**

| **LABORATORY TESTS** | | **Thrombotic Phenotype** | **Fibrotic Phenotype** | **P** |
| --- | --- | --- | --- | --- |
| **ADMISSION TESTS (Highest value)** | | |  |  |
|  | **Lactate dehydrogenase (U/L)*** | 422.11 ± 169.09 | 645.30 ± 77.88 | 0.09 |
|  | **C-reactive protein (mg/L)*** | 16.76 ± 9.45 | 12.60 ± 9.52 | 0.67 |
|  | **D-dimer (mg/L)*** | 13.26 ± 6.27 | 2.99 ± 1.628 | 0.02 |
| **DAILY TESTS (Highest value)** | |  |  |  |
|  | **White blood cells count (10³/µL)*** | 25.03 ± 10.36 | 27.18 ± 12.08 | 0.85 |
|  | **Hemoglobin (g/dL)*** | 11.99 ± 1.15 | 11.16 ± 1.04 | 0.24 |
|  | **Sodium (mmol/L)*** | 141.60 ± 6.23 | 137.50 ± 6.06 | 0.43 |
|  | **Potassium (mmol/L)*** | 5.19 ± 0.99 | 5.77 ± 0.94 | 0.30 |
|  | **Creatinine (mg/dL)*** | 3.57 ± 1.87 | 3.48 ± 1.10 | 0.99 |
|  | **Lymphocytes (10^3^/μl)*** | 2.5 ± 1.41 | 1.40 ± 1.31 | 0.37 |
|  | **Platelets (10^3^/μl)*** | 364.00 ± 129.07 | 225.60 ± 74.87 | 0.04 |
|  | **Neutrophils (10^3^/μl)*** | 20.44 ± 8.58 | 24.28 ± 10.47 | 0.70 |
| **LABORATORY TESTS (1, 3, and 5 days before death)** | | | |  |
| **One day before death** | |  |  |  |
|  | **White blood cells count (10³/µL)*** | 25.59 ± 11.29 | 27.23 ± 12.12 | 0.80 |
|  | **Hemoglobin (g/dL)*** | 10.31 ± 1.97 | 9.05 ± 1.59 | 0.36 |
|  | **Sodium (mmol/L)*** | 138.93 ± 7.40 | 131.25 ± 5.06 | 0.15 |
|  | **Potassium (mmol/L)*** | 4.80 ± 1.22 | 5.53 ± 0.90 | 0.28 |
|  | **Creatinine (mg/dL)*** | 3.47 ± 2.00 | 2.93 ± 1.12 | 0.68 |
|  | **Lymphocytes (10^3^/μl)*** | 2.04 ± 1.42 | 0.56 ± 0.21 | 0.01 |
|  | **Platelets (10^3^/μl)*** | 304.40 ± 125.35 | 190.97.31 | 0.09 |
|  | **Neutrophils (10^3^/μl)*** | 19.59 ± 10.06 | 23.94 ± 10.23 | 0.43 |
| **Three days before death** | |  |  |  |
|  | **White blood cells count (10³/µL)*** | 19.10 ± 8.15 | 16.42 ± 12.52 | 0.30 |
|  | **Hemoglobin (g/dL)*** | 11.12 ± 1.44 | 9.42 ± 1.18 | 0.52 |
|  | **Sodium (mmol/L)*** | 139.70 ± 6.23 | 136.56 ± 6.44 | 0.59 |
|  | **Potassium (mmol/L)*** | 4.79 ± 0.76 | 5.40 ± 1.37 | 0.45 |
|  | **Creatinine (mg/dL)*** | 2.03 ± 1.88 | 2.84 ± 2.05 | 0.95 |
|  | **Lymphocytes (10^3^/μl)*** | 1.19 ± 1.07 | 1.20 ± 0.70 | 0.92 |
|  | **Platelets (10^3^/μl)*** | 292.40 ± 81.96 | 188.20 93.23 | 0.05 |
|  | **Neutrophils (10^3^/μl)*** | 15.84 ± 6.85 | 14.16 ± 10.81 | 0.52 |
| **Five days before death** | |  |  |  |
|  | **White blood cells count (10³/µL)*** | 16.20 ± 6.13 | 16.36 ± 6.23 | 0.83 |
|  | **Hemoglobin (g/dL)*** | 10.85 ± 1.75 | 10.92 ± 1.45 | 0.91 |
|  | **Sodium (mmol/L)*** | 141.13 ± 5.93 | 138.06 ± 9.42 | 0.43 |
|  | **Potassium (mmol/L)*** | 4.51 ± 0.73 | 5.28 ± 0.58 | 0.07 |
|  | **Creatinine (mg/dL)*** | 2.59 ± 1.59 | 3.07 ± 1.34 | 0.51 |
|  | **Lymphocytes (10^3^/μl)*** | 0.90 ± 0.87 | 0.78 ± 0.32 | 0.55 |
|  | **Platelets (10^3^/μl)*** | 344.63 ± 154.60 | 205.20 ± 59.40 | 0.06 |
|  | **Neutrophils (10^3^/μl)*** | 14.91 ± 4.57 | 13.15 ± 5.18 | 0.92 |

**Note:** * mean ± standard deviation; (%) percentage.

**Supplementary** **Table S9 - Bimodal COVID-19 Phenotypes: Radiological Features.**

| **CHEST XR features** | | **Thrombotic Phenotype** | **Fibrotic Phenotype** | **P** |
| --- | --- | --- | --- | --- |
| **CHEST XR** | |  |  |  |
|  | **Ground-glass opacity (>50% area)** | 3 (30.0) | 4 (80.0) | 0.11 |
|  | **Pulmonary consolidation (%)** | 1 (10.0) | 1 (20.0) | 0.99 |
|  | **Pulmonary congestion (%)** | 1 (10.0) | 0 (0.0) | 0.99 |
|  | **Pleural effusion (%)** | 0 (0.0) | 2 (40.0) | 0.09 |
|  | **Interstitial opacities (>30% area)** | 5 (50.0) | 3 (60.0) | 0.99 |
|  | **Cardiomegaly (%)** | 6 (60.0) | 3 (60.0) | 0.99 |

**Note:** (%) percentage.

**SUPPLEMENTARY FIGURES**

**
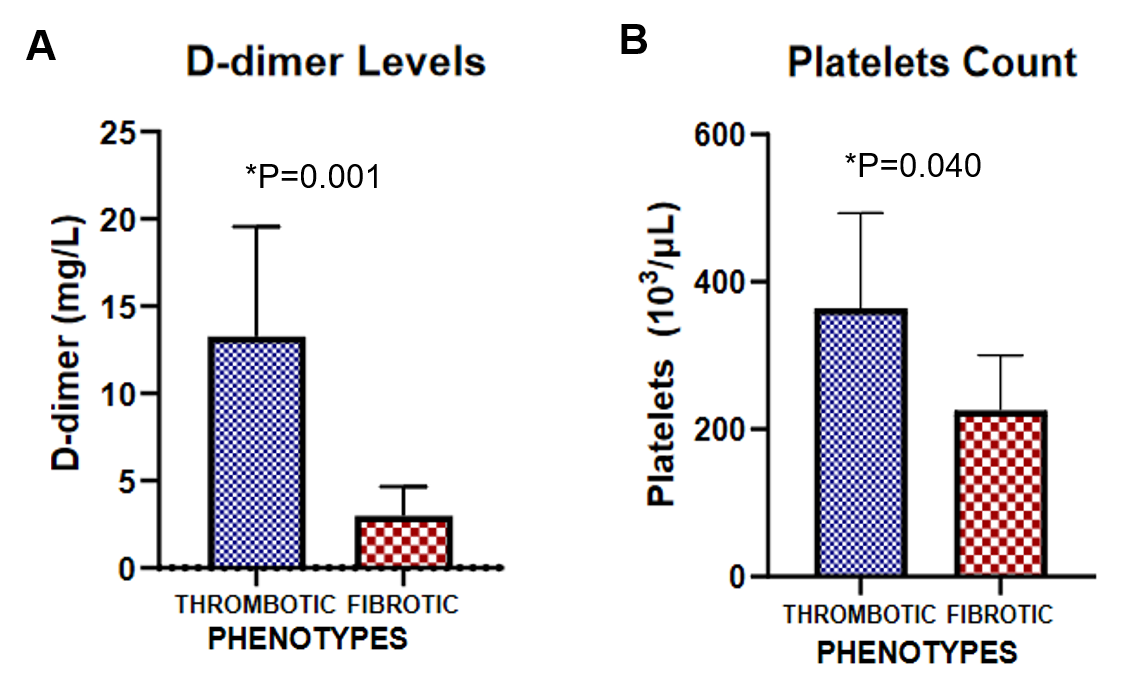
**

**Supplementary** **Figure S1 – Laboratory tests.** The thrombotic phenotype showed significant increased D-dimer levels (P=0.001) **(A)** and suggestive increased platelets count (P=0.040) compared to thrombotic one **(B)**.

**
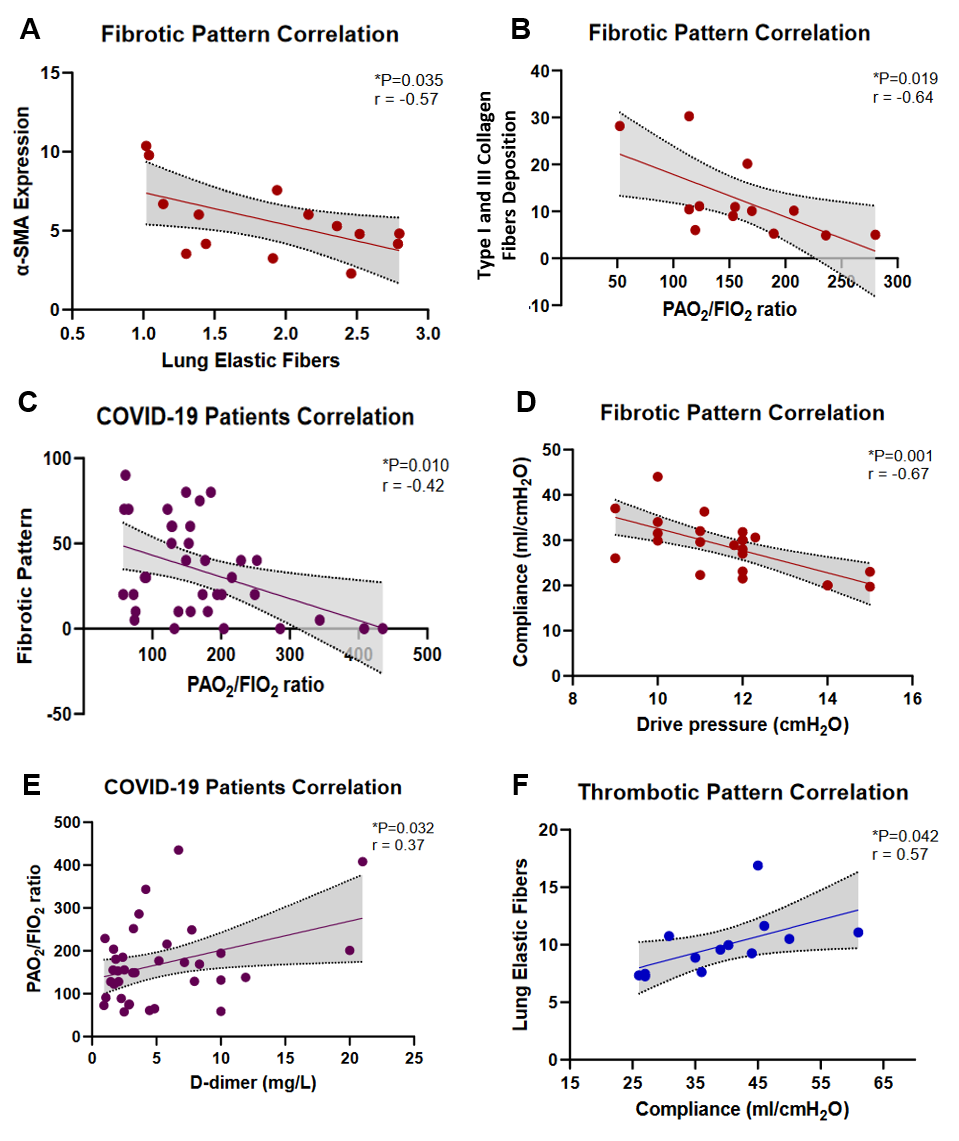
**

**Supplementary** **Figure S2 – Clinical and Pathological Correlations**. Following viral infection complications and subsequent mechanical ventilation, several pathophysiological processes occur that may lead to two opposite ends of lung injury, manifesting as two bimodal phenotypes. The fibrotic phenotype presented a remodeling lung injury after myofibroblasts activation, α-SMA expression, with further extracellular matrix deposition (collagen type I and III) that ultimately caused stiffness of the lung parenchyma, reducing elastic capacity (r=-0.57; P=0.035) **(A)** and impairing gas exchange (r=-0.64; P=0.019) **(B)**. This histological process was clinically demonstrated by progressive decline in PaO_2_/FiO_2_ ratio (r=-0.42; P=0.010) **(C)**. Additionally, the mechanical ventilation parameters analysis revealed that compliance values correlated negatively with drive pressure values for these patients (r=-0.67; P=0.001) **(D)**. On the other hand, the thrombotic phenotype clinically presented a progressive increase in PaO_2_/FiO_2_ ratio with lung injury recovery, but prominent thrombus formation confirmed by laboratory high D-dimer values (r=0.37; P=0.032) **(E)**. Due to the practically successful lung injury recovery, elastic fibers quantification presented normal values, consequently maintaining a regular compliance during mechanical ventilation support (r=0.57; P=0.042) **(F)**.

**
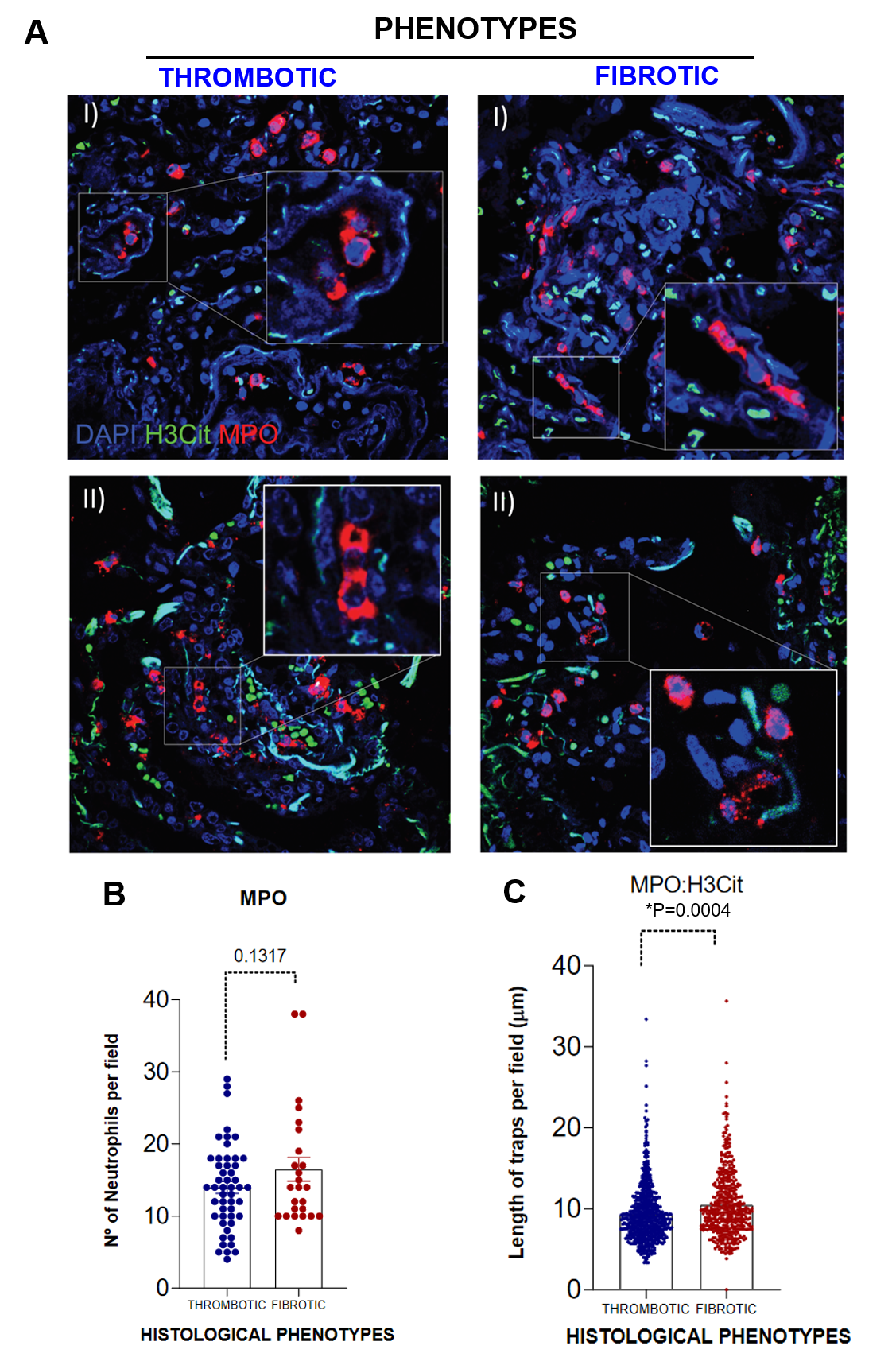
**

**Supplementary** **Figure S3 – Neutrophil-derived extracellular traps (NETs) in COVID-19 bimodal phenotypes.** Immunofluorescence identified neutrophils and NETs **(A)**. No significant quantitative difference of neutrophils between fibrotic and thrombotic phenotypes were found **(B)**. However, NETs were significantly higher in lung parenchyma from fibrotic phenotype than thrombotic one (P=0.0004) **(C)**.


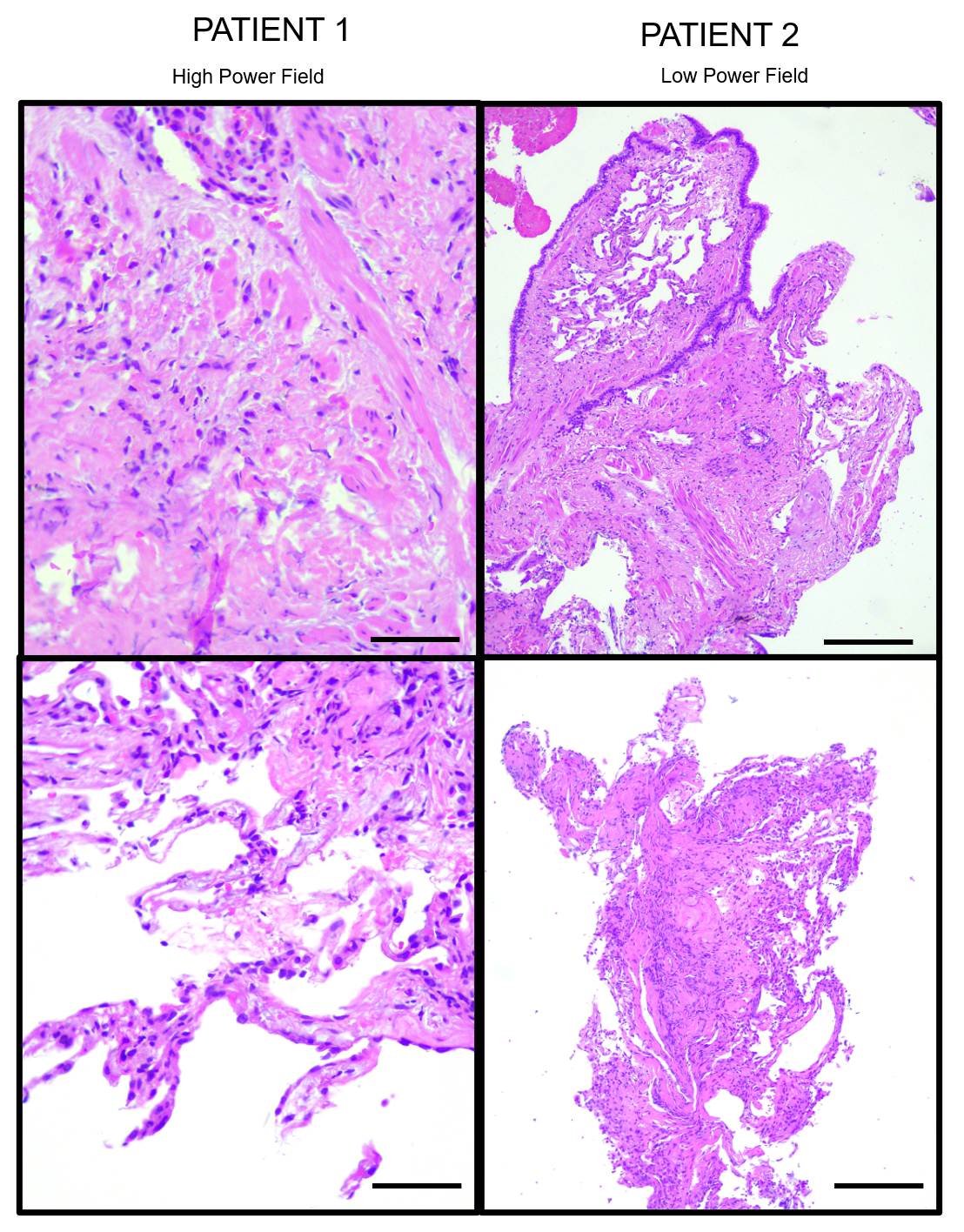


**Supplementary** **Figure S4 –** **Post COVID 19 Infection Transbronchial Biopsies Follow Up.** Two patients, both in their 60’s and male, ranging from three- to five-months’ post infection: There is minimal chronic inflammation and airway wall and lung parenchyma remodeling, characterized by submucosal scarring and alveolar septal thickening by fibrosis associated with alveolar pneumocyte prominence, respectively. While this may represent scarring secondary to resolving infection, the possibility of an evolving post infection chronic fibrosing disease needs to be excluded and more extensive studies are needed to investigate post COVID-19 complications in patients who survive the disease. Scale bar indicates 50 µm in high power field and 100 µm in low power field.
